# Tissue microarrays: applications in study of p16 and p53 alterations in Ewing's cell lines

**DOI:** 10.1186/1746-1596-3-S1-S27

**Published:** 2008-07-15

**Authors:** Rosa Noguera, Isidro Machado, Marta Piqueras, Jose Antonio Lopez-Guerrero, Samuel Navarro, Empar Mayordomo, Antonio Pellin, Antonio Llombart-Bosch

**Affiliations:** 1 Department of Pathology, University of Valencia, Valencia, Spain; 2 Fundación Instituto Valenciano de Oncología, Valencia, Spain

## Abstract

**Background:**

Tissue microarrays (TMAs) are used to study genomics and proteomics in several tumour tissue samples. Cell lines (CC) are of great importance in the study of the genetic changes in tumours, and some reveal several aspects of tumour oncogenesis. There are few published reports on Ewing's tumours with TMAs including original tumours (OT) and corresponding CC.

**Methods:**

We have performed four TMAs, from 3 OT and the corresponding CC of successive in vivo and in vitro tumour passages. Xenotransplant CC in nude mice from OT (XT/OT) was made. Subsequently multiple XT were performed and in vitro XT cell line (CC/XT) was obtained. In vivo re-inoculation of CC/XT (XT/CC) was planned. TMAs with the successive tumour passages that grew in nude mice (XT/OT and XT/CC) were analyzed by morphologic pattern (Hematoxilin/eosin), immunohistochemical staining (CD99, FLI1, p16, p53, ki-67), fluorescent in situ hybridization-FISH-(EWSR1 break apart, p16 and p53 status) and gene fusion types.

**Results:**

Heterogeneous results of the p16, p53 and ki67 in OT, XT/OT, CC/XT and XT/CC were observed. The three cell lines revealed EWS/FLI1 rearrangements. p16 gene was deleted only in one case. The deletion was detected by FISH and confirmed by PCR assays. A p53 alteration was found in the second case with monosomy and subsequently polysomic status of chromosome 17 during the evolution of CC. The PCR study revealed p53 mutation. The third case showed hypermethylation in the promoter of p16. The growth of the tumour in nude mice was more accelerated when the inoculation was performed from the CC/XT, increasing progressively over the passages. The third case did not reveal tumour growth in nude mice after the re-inoculation of CC/XT.

**Conclusion:**

The study of several cores from original tumours and successive tumour passages in TMAs facilitated the analysis of the genetic alteration and protein expression in Ewing's tumours.

## Introduction

Tissue microarrays (TMAs) are becoming the standard for the validation of prognostic biomarkers; this new technology leads to savings of time, cost and tissues [1]. TMAs consist of arrays of miniature core biopsy from several paraffin embedded tissue samples arrayed on a microscope slide, and are used to assess histopathological features, protein expression and fluorescent in situ hybridization (FISH) analysis [2-5]. There are few published reports on Ewing's sarcoma family tumours (ESFT) with TMAs including original tumours (OT) and corresponding xenograft and cell lines (CC).

ESFT is comprised of highly malignant bone and soft tissue tumours in children and young adults [6]. ESFT shares a common underlying molecular pathogenesis consisting of chromosomal translocations involving the EWS gene and one of the members of the ETS family of transcription factors. The best approach in ESFT is based in the integration of clinical data, histopathology, immunohistochemistry (IHC), electron microscopy, FISH and molecular diagnostic technologies [6-10]. The CC is of great significance in the study of the principal aspects of the tumour oncogenesis and in several cases is able to reveal the genetic changes of these tumours [11,12]. Several ESFT cell lines are available and they have been established and characterized using cytogenetic and molecular biology studies isolated usually from the OT and on a few occasions from the in vivo and in vitro CC cultures [11,12]. The aims of this report are the analysis of p16 and p53 of three Ewing's cell lines using TMA assays, and the possible relation between p16 and p53 alterations and growth velocity (Ki-67) of xenotransplanted tumours (XT): original tumour xenotransplants and in vitro culture xenograft.

## Materials and methods

### Samples sources

The clinical data of the three patients are show in Table 1.

**Table 1:** Clinical data.

	**URE RA (PT 455)**	**SAN VI (PT450)**	**PAD AN (PT 445)**
**Age/Sex**	17 years/Male	15 years/Male	16 years/Male
**PrimarySite**	Right Scapula	Dorsal Raquis	Maleolar region of left foot
**Recurrence**	no	yes	multiple
**Metastasis**	yes (liver and lung)	no	yes (lung)
**Treatment**	Surg+Chemo+Radio	Surg+Chemo+Radio	Surg+Chemo+Radio
**Status**	Died	Died	Died

### Methodology

Initially, in vivo cell culture (xenotransplant from original tumour-XT/OT) was performed. Subsequently multiple xenotransplanted were performed and XT tumour cell line in vitro (CC/XT) was obtained. OT, XT/OT and CC/XT were analyzed and characterized by morphologic pattern, IHC, FISH and gene fusion types. Subsequently, in vivo re-inoculation of these cell lines in nude mice (XT/CC) was performed and all studies were made.

### Assembly of TMA

Four TMAs were constructed; three included OT and the corresponding XT. The passages in nude mice were divided into initial (passages 1–7), middle (passages 8–16) and final (passages 20–30) to group the histopathological and IHC features of the XT. The last TMA was performed with tumours from XT/CC, in these cases the passages were divided into initial (passages 1–5), middle (passages 6–10) and final (passages 11–16). TMA construction was performed as described previously [13,14].

### Immunohisto-cytochemical analysis

IHC analysis was performed using anti-CD99 antibody (clone 12E7, DakoCytomation) at a 1:50 dilution, anti-p16 antibody (clone F12, Santa Cruz Biotechnology, Santa Cruz, CA) at 1:100 dilution, anti-p53 antibody (clone DO7, Novocastra) at 1:50 dilution, anti-Ki-67 antibody (MIB-1, DakoCytomation) at 1:50 dilution, anti-FLI-1 polyclonal antibody at 1:25 dilution, the rest of methodology was performed as described [13]. The nuclear staining was considered positive for p53, p16, Ki-67 and FLI-1 antibodies, cytoplasmic staining was not scored as a positive phenotype. Sections were examined and immunoreactivity was defined as follow: (-): <5%; (+): 5% to 10%; (++):10% to 50%; (+++): >50%.

### Xenotransplant

Male nude mice, were purchase from IFFA-CREDO (Lyon, France), kept under specific pathogen-free medium throughout the experiment, and provided with vinyl isolates plus sterilized food, water, cage and bedding. The specimens for xenotransplant were obtained at surgery (OT) and placed in (RPMI 1640) medium plus antibiotic at 37 ℃ until transplantation, frequent 6 hours after surgery. Fragments of non-necrotic tumour about 3 to 5 mm in size were transplanted into the subcutaneous tissue in the backs of the nude mice (XT/OT). The new tumour transfers were made by following the same procedure as in the initial xenotransplant as previously described [13], and always under highly sterile conditions. Two animals were used in each passage. Materials from different passages were obtained in order to perform all TMAs.

### Cultures and cytogenetic studies

In order to perform in vitro CC as well as cytogenetic analysis, the OT and XT/OT were placed in RPMI 1640 medium at 4 ℃, immediately after removal from the patient or from the nude mice, and transferred to the molecular pathology laboratory as soon as possible. The material was cultured as previously described [10-12].

### FISH analysis

Section were processed for FISH using the EWSR1 (22q12) dual colour (red-green), break apart probe (Vysis), p16 (CDKN2A) gene specific probe (9p21-red)/abl gene (9q34) (green) as control and p53 gene specific probe (red)/MPO gene (17q23) green as control. FISH was performed according to the conventional protocol for cytology preparation and paraffin tumour[13,14]. At least 100 non-overlapping tumour cells were evaluated for each hybridization for three different authors (RN, IM and MP). Cells with presence of translocation involving EWS, one fused or very near (red-green) signal and the other signals split into one green and one red signal will be observed. In normal cells with no translocation, two fused or very near (red-green) signal will be expected. A positive result was defined as more than 15–20% of cells with translocation. In the case of p16 analysis the results will be according to the following criteria: no deletion (cells with the same number of red and green signals), gain (cells with more red signals than green signals), homozygous deletion (cells with 2 green signals and no red signals), heterozygous deletion (cells with 2 green signals and 1 red signal), and heterozygous/homozygous deletion (combination of cells with 2 green signals and not red signals plus cells with 2 green signals and 1 red signal). The remaining signal combinations in cells represent a possible cut nuclei artefact. A positive result was defined as more than 15% of cells having deletions. In case of metaphases from in vitro CC the aberrations were easily detected.

### Molecular biology

We performed RT-PCR on tumours to assess the presence of EWS/FLI-1, EWS/FEV, EWS/ERG fusion products as well as p16 and p53 status according to standard protocols [10,13,14].

## Results

CD99 and FLI-1 expression was strong (+++) in all samples. FISH analysis revealed a positive translocation (EWS break apart) in the three cells lines and the fusion genes was EWS/FLI-1 in all the cases. The immunohisto-cytochemical, FISH results, and PCR study of p16 and p53 in OT, XT/OT, CC/XT and XT/CC as well as proliferation index (KI-67) are as follows:

### First case (Table 2)

**Table 2:** PT455

**Sample**	**IHC p16**	**FISH p16**	**PCR p16**	**IHC p53**	**FISH p53**	**PCR p53**	**Ki-67**
**OT**	+++	Hm/Ht deletion 2:0(25%), 2:1(25%)	P16-1 and P16-2 deleted	-	No deletion	No mutation	-
**XT/OT (Nu199) (P1–P7)**	+++	Hm/Ht deletion 2:0(25%), 2:1(20%)		-	No deletion		-
**(P8–P16)**	+++	Hm/Ht deletion 2:1(60%)		-	No deletion	No mutation	-
**(P20–P30)**	+++	NV		-	No deletion		-
**CC/XT**	+++	Hm/Ht deletion 2:1(80%)		-	No deletion		-
**XT/CC (Nu432) (P1–P5)**	+++	Hm/Ht deletion 2:0(60%)		++	No deletion		+++
**(P6–P10)**	+++	Hm/Ht deletion 2:0(85%)		++	No deletion		+++
**(P11, P13–P16)**	+++	Hm/Ht deletion 2:0(90%)		+++	No deletion		+++

The histopathological diagnosis of OT was atyical Ewing sarcoma with large cells, p16 IHC staining revealed strong positivity and by FISH heterozygous/homozygous deletion. PCR study confirmed p16 deletion. p53 was negative by IHC and FISH showed neither deletion nor polysomy. XT/OT and CC/XT maintained the same characteristics of OT concerning p16 and p53 expression as well as FISH results. Nevertheless in XT/CC, p53 expression was strongly positive and the percentage of p16-deleted cells was higher than in OT and all other samples. The growth velocity of nude tumour was higher after the re-inoculation (XT/CC) and the proliferation index (Ki-67) expression in XT/OT was negative.

### Second case (Table 3)

**Table 3:** PT450

**Sample**	**IHC p16**	**FISH p16**	**PCR p16**	**IHC p53**	**FISH p53**	**PCR p53**	**Ki-67**
**OT**	-	No deletion	normal	-	Monosomy	Mutation 151:CCC/CGC Prox/Arg	+
**XT/OT (Nu267) (P1–P7)**	-	No deletion		++	Polysomy 3:3(70%)		+
**CC/XT**	-	No deletion		+++	Polysomy 3:3(75%)		+
**XT/CC (Nu416) (P1–P5)**	3+	No deletion		+++	Polysomy ++ 3:3(80%), 4:4, 6:6 (5%)		+++
**(P6–P8)**	3+	No deletion		+++	Polysomy ++ 3:3(85%), 4:4, 6:6 (5%)		+++
**(P9–P11)**	3+	No deletion		+++	Polysomy +++ 3:3(85%), 4:4, 6:6 (15%)		+++

The histopathological diagnosis of OT was classical Ewing sarcoma, p16 IHC staining was negative and by FISH revealed no deletion. p53 was negative by IHC and FISH showed no deletion but did show monosomy. PCR study revealed a mutation in p53 gene. XT/OT showed no variation in p16 expression, although p53 expression was moderately positive (++) and p53 polysomy was detected by FISH. p16 FISH study was similar in CC/XT and XT/CC but p53 expression was higher with more polysomy of chromosome 17 detected by FISH. The growth velocity of nude tumour was higher after the re-inoculation (XT/CC); the proliferation index (Ki-67) expression in XT/OT was weakly positive and very high in XT/CC.

### Third case (Table 4)

**Table 4:** PT445

**Sample**	**IHC p16**	**FISH p16**	**PCR p16**	**IHC p53**	**FISH p53**	**PCR p53**	**Ki-67**
**OT**	+++	No deletion	Methylation altered	-	No deletion	No mutation	-
**XT/OT (Nu22) (P1–P7)**	++	No deletion		+	No deletion		-
**(P8–P16)**	+++	No deletion		+	No deletion		-
**(P20–P30)**	-	NV		+	No deletion		-
**CC/XT**	-	No deletion		-	NV		-
**XT/CC (Nu441)**	No growth	No growth	No growth	No growth	No growth	No growth	No growth

The histopathological diagnosis of OT was atypical Ewing sarcoma. p16 and p53 showed no deletion neither polysomy by FISH in OT, XT/OT and CC/XT. PCR revealed p16 methylation and no alteration in p53. After re-inoculation (XT/CC) the tumour did not grow in nude.

## Discussion

Many research laboratories are taking great advantage of TMAs in biomarker validations and study of the biology of several tumours, furthermore, TMAs enable the preservation of tissue banks and their construction is not difficult. Currently, TMAs are becoming as indispensable tool for translating laboratory findings to the clinic [1-3,15]. In vivo and in vitro Ewing's cell lines remain an invaluable research tool in the study of the multistep progression of ESFT. The combination of TMAs, xenograft in nude mice and in vitro cell line studies in ESFT enable researchers to determine the clinical significance of specific morphological and protein alterations in several tumours samples and facilitate a comparison of protein expression and histopathological variation in successive tumours passages [5,13,14]. The analysis of OT and xenograft tumours were performed using TMA analysis in order to discover the variations in histopathological, tumour heterogeneity and progression, and immunohistochemical profile. We study the expression profile using triplicate tissue cores because they offer a high concordance rate with whole sections. Several studies on tumour progression in different tumours and cell lines using TMAs have been published [13,16] but there are few studies involving TMAs, cell lines and xenografts in ESFT. The clinical data in the three cases were similar with scant variation as the ESFT cases described in the literature [6,10,17]. Morphologically, the most interesting change was the transition of most of the primary tumours to a more undifferentiated tumour in the xenograft similar to the features described by other authors in sarcoma xenograft study [13,16], although the IHC profile revealed strong expression of CD99 and FLI-1 antibodies in all the OT, cell lines and most of the xenotransplanted tumour. A slight decrease in CD99 expression was observed in terminal passages in xenograft but was not significant. EWS break-apart was positive as a reflex of EWS/FLI-1 gene fusion as the most frequent genetic aberration described in ESFT [6,10,12,18,19]. p16 and p53 alterations are reported to be important in tumour progression, both are the most frequent alterations relate to cancer [8,12,20-26]. Hypermethylation is a powerful mechanism for the silencing of tumour suppressor genes in cancer, but intriguingly, only the two cases with genetic alteration developed growth in nude after re-inoculation of XT/CC, the case with epigenetic alteration did no grow in nude after re-inoculation. The high index of growth in XT/CC could be related with a higher percentage of cells with genetic and/or epigenetic alterations. The stroma cells or necrotic cells in OT could limit the velocity of growth of the tumour in nude after xenograft. Therefore, in culture, Ewing's tumour cells grow very fast and replace stroma and necrotic cells, then after inoculation from cell culture, the possibility of growth in nude is higher. The tumour cells in culture could acquire new genetic alterations and/or aberrant protein expressions that improved the aggressive phenotype. In fact our XT/CC showed new aberrant p16 and p53 expression as well as increased genetic alterations. Milne AN et al. [16], described recently that epigenetic changes may represent alterations present in more aggressive subclones or could be induced during tumour progression or cell lines establishment.

## Conclusion

The use of TMA-based morphological and immunohistochemical evaluation of Ewing's sarcoma proved to be an important tool in demonstrating the phenotypic variations of xenografted Ewing's sarcoma. The implementation and analysis of several cores in OT and successive passages from xenograft enable us to describe the histopathology and phenotypic variations in theses tumours samples.
